# The alterations in the gut microbiota and *Bifidobacterium animalis* BD400 during the development and progression of collagen-induced arthritis in rats

**DOI:** 10.3389/fimmu.2026.1821878

**Published:** 2026-09-18

**Authors:** Yang Yang, Qing Hong, Zhenmin Liu

**Affiliations:** 1 State Key Laboratory of Dairy Biotechnology, Engineering Research Center of Dairy Biotechnology, Shanghai, China; 2 Key Laboratory of Functional Dairy Products Processing, Ministry of Agriculture and Rural Affairs, Shanghai, China; 3 Dairy Research Institute, Bright Dairy & Food Co., Ltd., Shanghai, China

**Keywords:** 16S rRNA, *Bifidobacterium animalis*, development and progression, gut microbiota, rheumatoid arthritis

## Abstract

Rheumatoid arthritis (RA) is a systemic, chronic autoimmune disease. Based on the previous research results we conducted on the prevention of RA with *Bifidobacterium animalis* BD400, we generated a large longitudinal 16S rRNA sequencing dataset to characterize temporal microbial shifts associated with disease progression and probiotic intervention. This study constructed collagen-induced arthritis (CIA) model rats to assess the changes in the gut microbiota species of the rats during the model development. The experimental results showed that as the CIA model developed, the gut microbiota of the rats underwent dynamic changes involving genera such as *Akkermansia*, *Romboutsia*, *Christensenellaceae*_R-7_group, *Bifidobacterium*, *Turicibacter*, *Candidatus*_*Saccharimonas*, etc. *Bifidobacterium animalis* BD400 administration altered the relative abundances of genera such as *Turicibacter*, *Bifidobacterium*, *Lactobacillus*, *Lacticaseibacillus*, and *Ligilactobacillus*, thereby helping to alleviate RA. This study provides data reference for achieving RA prevention through gut microbiota intervention and lays the foundation for the application of probiotics in RA prevention.

## 1 Introduction

Rheumatoid arthritis (RA) is a chronic autoimmune disorder driven by persistent synovial inflammation, progressive articular hyperplasia, and irreversible cartilage and bone destruction. Globally, RA affects 0.25%–1% of the population (1), while its prevalence in the Chinese population reaches 0.42%, corresponding to roughly 5 million diagnosed patients with a marked female predominance (male:female ≈ 1:4) (2). Although RA can manifest across all age groups, disease onset rises sharply after the age of 40 (3). Current clinical interventions primarily target inflammatory suppression and slow joint deterioration; no existing pharmacotherapy is capable of reversing established cartilage erosion. The multifactorial, incompletely defined etiology of RA continues to hinder the development of curative treatments.

Gut microbiota is now recognized as a critical environmental regulator of systemic autoimmunity and is tightly implicated in RA pathogenesis throughout disease initiation and progression (4, 5). However, longitudinal shifts in gut microbial communities alongside advancing RA pathology, as well as the core taxonomic genera governing disease severity, remain poorly characterized. Targeted probiotic supplementation has emerged as a promising gut-intervention strategy for mitigating RA symptoms. *Bifidobacterium animalis* is a core commensal bacterium in mammalian and human intestines that participates in intestinal nutrient digestion, mucosal barrier maintenance, and systemic immune homeostasis (6, 7). Well-characterized subspecies (e.g., BB-12, HN019) exhibit robust acid and bile salt resistance, enabling stable intestinal colonization; these strains are generally recognized as safe for vulnerable populations including infants, pregnant individuals, and immunocompromised groups (8). Multiple *Bifidobacterium* strains have demonstrated protective effects against collagen-induced arthritis (CIA, the gold-standard preclinical RA model): five *B. adolescentis* isolates alleviate CIA clinical manifestations and restore immune homeostasis *via* early intervention (9), whereas *B. longum* RAPO suppresses pro-inflammatory IL-17 secretion to reduce arthritis incidence, joint inflammation, and bone/cartilage damage in CIA mice (9). Notably, direct evidence supporting RA-modulating efficacy of *B. animalis* strains remains extremely limited, representing a key research gap.

Our previous complementary *in vivo* work (10) validated the anti-arthritic capacity of *B. animalis* BD400 in CIA rats. Oral BD400 treatment significantly reduced clinical arthritis scores and paw edema, alleviated knee osteochondral fusion and cartilage calcification, and suppressed intra-articular pro-inflammatory TNF-α and matrix-degrading MMP-13. Consistently, BD400 lowered serum anti-collagen II autoantibodies (IgG, IgG1, IgG2a), circulating IL-17A, and synovial TNF-α/IL-1β, while upregulating intestinal tight-junction transcripts (occludin-1, MUC-2, ZO-1) to reinforce mucosal barrier integrity. Metabolomic profiling further revealed BD400 reshapes histidine metabolism by reducing 1-methyl-L-histidine and urocanate, offering a partial mechanistic explanation for its anti-arthritic activity. Although our prior study confirmed that BD400 reshapes the static phylum- and genus-level gut microbiota, the weekly longitudinal fecal microbial samples collected throughout that trial were not previously analyzed, leaving unresolved how BD400 dynamically remodels gut microbial succession over the full course of CIA progression.

Against this backdrop, the present study extends our earlier phenotypic and metabolomic findings by systematically interrogating time-resolved gut microbiome dynamics in CIA rats with or without BD400 intervention. We aimed to characterize longitudinal shifts in α- and β-diversity across disease progression, identify unique and shared amplicon sequence variants (ASVs) among experimental groups, and pinpoint differential taxonomic signatures at phylum and genus levels modulated by BD400. We further contextualize microbial alterations relative to CIA disease phenotypes, address compositional data limitations of 16S sequencing, and discuss key microbial biomarkers with translational relevance to early CIA. Collectively, this longitudinal microbiota analysis clarifies how *B. animalis* BD400 remodels gut microbial trajectories to counteract CIA-linked dysbiosis, filling critical knowledge gaps regarding dynamic probiotic-microbe-autoimmunity crosstalk.

## 2 Materials and methods

### 2.1 Materials

Bovine type II collagen (CII) solution, complete Freund`s adjuvant and incomplete Freund`s adjuvant were purchased from Chondrex (Redmon, WA, USA). Methotrexate (MTX) was purchased from Shanghai Yuanye Bio-Technology Co. (Ltd, Shanghai, China).

### 2.2 Bacterial strain

All *Bifidobacteria* were cultured overnight in MRS medium supplemented with 0.5% L-cysteine at 37 °C. The cultures were centrifuged at 3000 × g for 10 min at 4 °C to harvest the bacterial cells. The cell pellet was then washed twice with sterile saline. The cells were subsequently resuspended in sterile saline and subjected to viable cell counting. The bacterial suspension was stored at -80 °C until use. Before each daily intragastric administration, the bacterial suspension was resuspended in sterile saline at 2 × 10^8^ CFU/mL for daily administration to the rats.

### 2.3 Experimental animals

Female Wistar rats aged 7–8 weeks were housed in a specific pathogen-free environment at a temperature of 25 ± 2°C and a humidity of 50% ± 5%. They were maintained under a 12-hour light-dark cycle. Food and water were provided ad libitum. The rats were allowed to acclimatize for 7 days before the experiment. All animal experiments were conducted in accordance with the “Regulations on the Management of Laboratory Animals in China”. The protocol of this study has been approved by the Institutional Animal Care and Use Committee of Shanghai Rat Biotechnology Co., Ltd. (Approval No: 202306261).

### 2.4 Establishment of CIA rats and experiment design

The Wistar rats were randomly divided into 4 groups, with 7 rats in each group: the control group, CIA group, MTX group, and *Bifidobacterium animalis* BD400 group (abbreviated as BD400 group). The CIA, MTX, and BD400 groups were subjected to CIA modeling. The method for establishing the CIA rat model referred to a previous study (11). Briefly, bovine CII (2 mg/mL) was dissolved in 0.05 M acetic acid and emulsified in an equal volume of complete Freund’s adjuvant, and 0.15 mL of the emulsion was injected subcutaneously at the base of the tail as the first immunization. Seven days later, an equal volume of CII emulsified in incomplete Freund’s adjuvant was injected subcutaneously at the base of the tail, avoiding the primary injection site, as the booster immunization.

MTX group: after the first immunization, MTX (7.6 mg/kg/week) was used as the standard drug, administered twice a week, and sterile saline was intragastrically administered at other times. Rats in the *Bifidobacterium animalis* BD400 group were orally administered the bacterial suspension (2 × 10^8^ CFU/mL) daily. Control group: rats received sterile saline by intragastric administration (non-CIA, non-immunized rats with saline gavage only). The experiment lasted for 56 days, including a 7-day adaptation period. The experimental schedule is shown in **Figure 1**. Rat feces were collected every 7 days.

**Figure 1:**
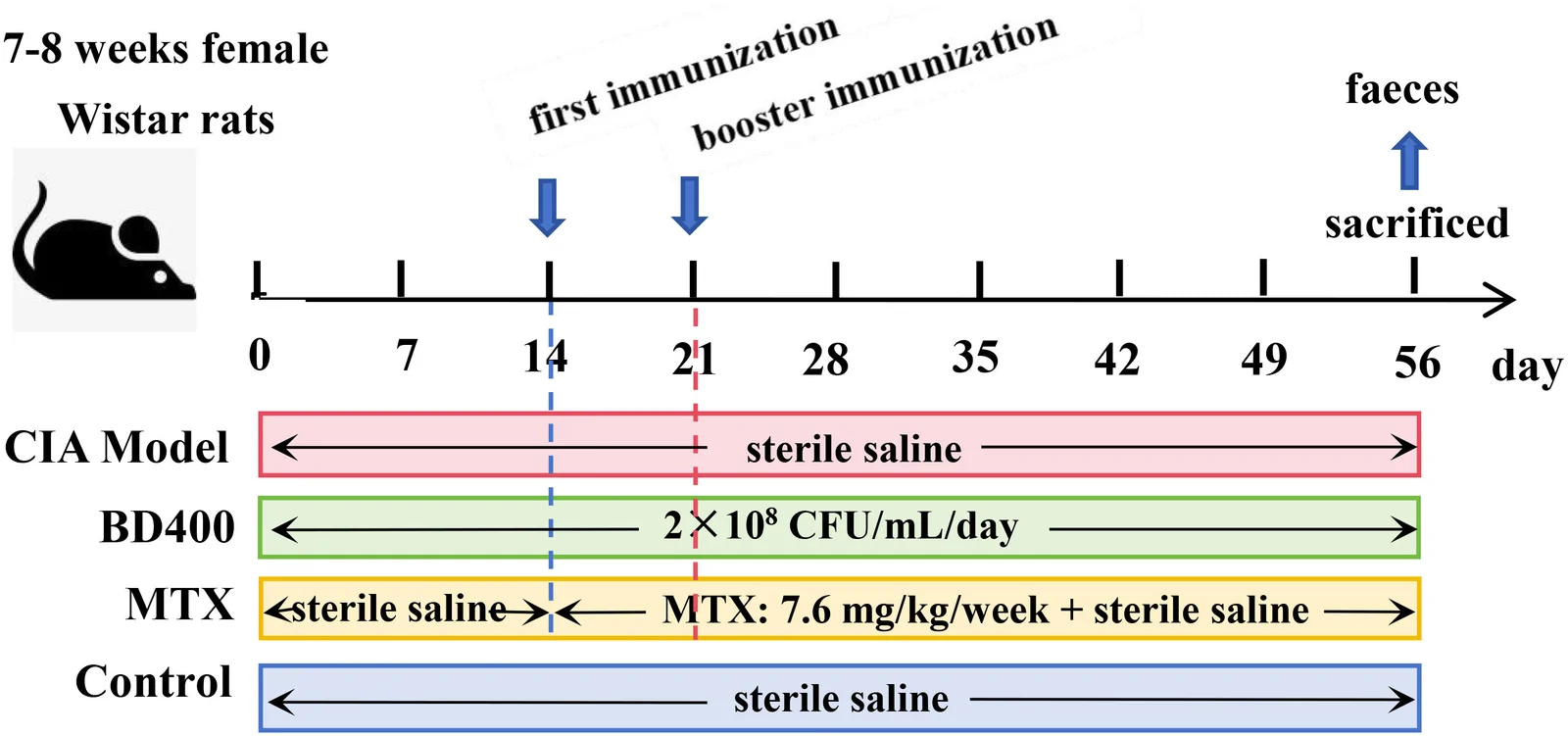
Experimental schedule.

### 2.5 Analysis of the microbial community

Fecal samples were homogenized and diluted 10-fold with sterile phosphate-buffered saline (PBS) to obtain uniform microbial suspensions. DNA was extracted from fecal samples using the FastDNA Spin Kit (Cat. No. 116570200). The diluted DNA was used as the template for PCR amplification of the 16S rDNA V3-V4 region. The V3–V4 hypervariable regions of the bacterial 16S rRNA gene were amplified by PCR using the primer pair 341F/805R (forward: 5′-CCTACGGGNGGCWGCAG-3′; reverse: 5′-GACTACHVGGGTATCTAATCC-3′). The PCR amplification mixture consisted of 25 μL 2 × Premix Taq, 2 μL each of forward and reverse primers (10 μM), 1 μL of genomic DNA template, and 20 μL of sterile double-distilled water. The size of the PCR amplification product bands was detected by 1% agarose gel electrophoresis. The target band was approximately 465 bp. The target band of 450–500 bp size was excised with a sterile scalpel and recovered. The concentration of the purified PCR product was determined using a fluorescence quantification kit (Quant-iT PicoGreen dsDNA assay kit, P11496) according to the manufacturer’s instructions. The amplified products are purified using a 1% agarose gel extraction kit. The purified samples are quantified using Qubit 2.0, normalized to an equimolar concentration, pooled for library preparation, and sequenced on the Illumina MiSeq platform according to the manufacturer’s instructions.

Raw paired-end 16S rRNA gene sequences were demultiplexed and quality-filtered using QIIME2 and DADA2 pipeline (12). Reads were truncated at a quality threshold of Q2, and sequences with maximum expected errors (MaxEE) greater than 2 were discarded. Reads containing ambiguous bases (N) were completely removed. The truncated length was set to 240 bp for both forward and reverse reads. After paired-end merging, sequence denoising, error correction, and chimera removal were performed to generate amplicon sequence variants (ASVs) with single-nucleotide resolution. Contaminant sequences corresponding to mitochondria and chloroplasts were eliminated, and singleton ASVs with extremely low abundance were filtered out to reduce technical noise. Taxonomic annotation of qualified ASVs was performed against the SILVA 138.2 SSU Ref NR 99 database at domain, phylum, class, order, family, and genus levels. Species-level classification was not adopted due to the limited resolution of short-read 16S amplicons.

### 2.6 Statistical analysis

The statistical analysis was conducted using SPSS Statistics 30.0.0, with *P* < 0.05 considered significant. Graphs were drawn using R-based visualization tools and GraphPad Prism (10.4.1). For alpha diversity analysis, community richness indices (Chao1) and diversity indices (Shannon, Simpson) were calculated, and inter-group differences were statistically analyzed using the Wilcoxon test. Beta diversity was evaluated based on Bray-Curtis, Jaccard, and weighted UniFrac distance matrices. Principal coordinate analysis (PCoA) was performed to visualize microbial community structural differences across groups. Permutational multivariate analysis of variance (PERMANOVA) and analysis of similarities (ANOSIM) were conducted to quantitatively assess the degree of community structural variation among different groups. The taxonomic composition characteristics of the microbial community at the phylum and genus levels are presented through Sankey plots, and line plots of genus-level abundance was used to visualize the distribution of different microorganisms over time. The LEfSe analysis is employed to identify the differential microbial taxa between the groups (LDA score > 3.0 and *P* < 0.05). Linear mixed-effects models with restricted maximum likelihood (REML) estimation were constructed to quantify the temporal and intergroup variations of gut microbiota. Fixed factors included Group, Time, baseline covariate Day 0, and Group×Time interaction; random intercepts of individual ID were incorporated to account for repeated measurements, and compound symmetry (CS) covariance structure was applied for within-subject longitudinal data. Least significant difference (LSD) correction was adopted for *post-hoc* pairwise comparisons of simple effects.

## 3 Results

### 3.1 Sequencing data validation and ASV dynamic analysis

Fecal samples collected weekly from different groups were subjected to 16S rDNA high-throughput sequencing to assess the changes in the gut microbiota during CIA progression, as well as the differences between the various groups. The paired-end data were merged, and quality control and chimeric sequence filtering were performed. A total of 13,240,066 valid sequences were obtained. Rank-abundance curves, rarefaction curves and ASV accumulation curves were applied to evaluate the sequencing quality, species richness and sampling adequacy of 16S rRNA gene sequencing data (**Supplementary Figure 1**). The results of rat arthritis scores and paw swelling were presented in **Supplementary Material Figure 2**.

Petal Venn diagrams were constructed to quantify shared ASVs and timepoint-exclusive ASVs across eight serial sampling days (Day 0–Day 56) within each group, revealing longitudinal shifts of gut microbial ASVs under distinct treatments. The control group (**Figure 2A**) possessed the largest number of shared ASVs (474) throughout the entire experimental period, whereas the CIA model group (**Figure 2B**) harbored fewer shared ASVs (448). The maximum number of unique ASVs at single time points in the control group reached 2823 (Day 42), which was markedly higher than the peak unique ASVs of the CIA model group (2573 at Day 0). These data indicated that CIA modeling reduced the overall temporal turnover of ASVs and disrupted the stability of the gut microbiota. MTX intervention further reduced the number of shared ASVs to 420 (**Figure 2C**), the lowest shared ASV count among all four groups, compared with 448 shared ASVs in the CIA model group. The range of timepoint-specific ASVs in the MTX group was narrowed to 2008–2472, with lower peak unique ASVs than the CIA model group. This observation suggested that MTX treatment aggravated the loss of intestinal ASVs based on the dysbiotic microbiota induced by CIA induction. The BD400 group (**Figure 2D**) retained 473 shared ASVs, nearly equivalent to the control group (474) and substantially higher than both CIA model (448) and MTX groups (420). The range of timepoint-unique ASVs in the BD400 group spanned 2031 (Day 28) to 2667 (Day 14), showing abundant temporal microbial turnover comparable to healthy controls. Collectively, BD400 supplementation effectively preserved the integrity of the gut microbiota against CIA-induced dysbiosis, and avoided the gut microbiota depletion observed in the MTX group.

**Figure 2:**
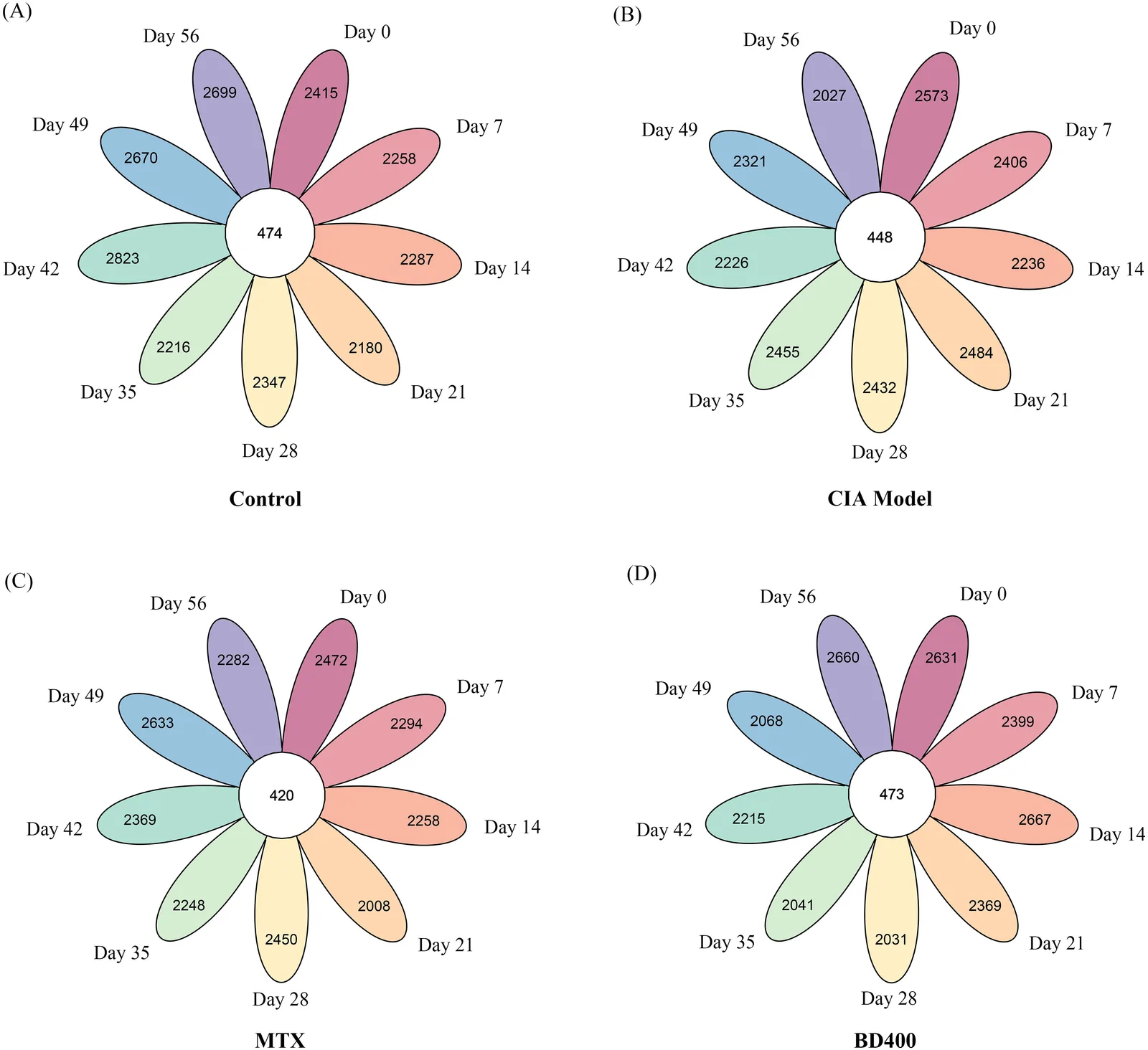
Petal Venn diagrams showing shared ASVs and timepoint-specific unique ASVs within each treatment group. **(A)** Control group; **(B)** CIA model group; **(C)** MTX group; **(D)** BD400 group. Numbers in the central circle represent shared ASVs detected at all time points within the group, and values on each petal represent ASVs exclusively identified at the corresponding sampling day.

### 3.2 Longitudinal analysis of gut microbial α diversity

Longitudinal dynamic changes in three classic α-diversity indices (Chao1, Shannon, Simpson) of gut microbiota were tracked throughout the experimental period to evaluate the effects of CIA modeling and different interventions on gut microbial richness and diversity (**Figure 3**). The control group maintained steadily high Chao1, Shannon and Simpson indices across all time points, reflecting stable, high gut microbial richness and diversity under healthy conditions. In contrast, the CIA model group exhibited sustained reductions in all three α-diversity metrics after CIA induction. Notably, significant declines were observed at Day 14 for Chao1 index (*P* < 0.05) and Shannon index (*P* < 0.05) relative to the control group. These results demonstrated that CIA arthritis modeling markedly disrupted gut microbial homeostasis by reducing both the species richness and community diversity of gut microbiota. Compared with the CIA model group, MTX treatment triggered a more severe drop in α-diversity at Day 21: the MTX group displayed the lowest Chao1, Shannon and Simpson values at this time point, with a statistically significant reduction in Simpson index at Day 21 (*P* < 0.05). Although α-diversity partially recovered in the late stage, the overall α-diversity level of the MTX group remained inferior to the CIA model group throughout most time points. This indicated that MTX monotherapy further aggravated the loss of gut microbial richness and diversity on the basis of CIA-induced dysbiosis. The BD400 group exhibited consistently elevated Chao1, Shannon and Simpson indices compared with the CIA model and MTX groups over the whole experimental timeline. At Day 63, the Chao1 index of the BD400 group was significantly higher than that of the CIA model group (*P* < 0.01). The temporal fluctuation amplitudes of all three α-diversity indicators in the BD400 group were minimal, and its overall diversity profile was highly comparable to the healthy control group. Collectively, BD400 supplementation effectively restored and sustained high gut microbial richness and diversity, counteracting the α-diversity loss caused by CIA induction and avoiding the severe microbiota diversity depletion observed in the MTX group.

**Figure 3:**
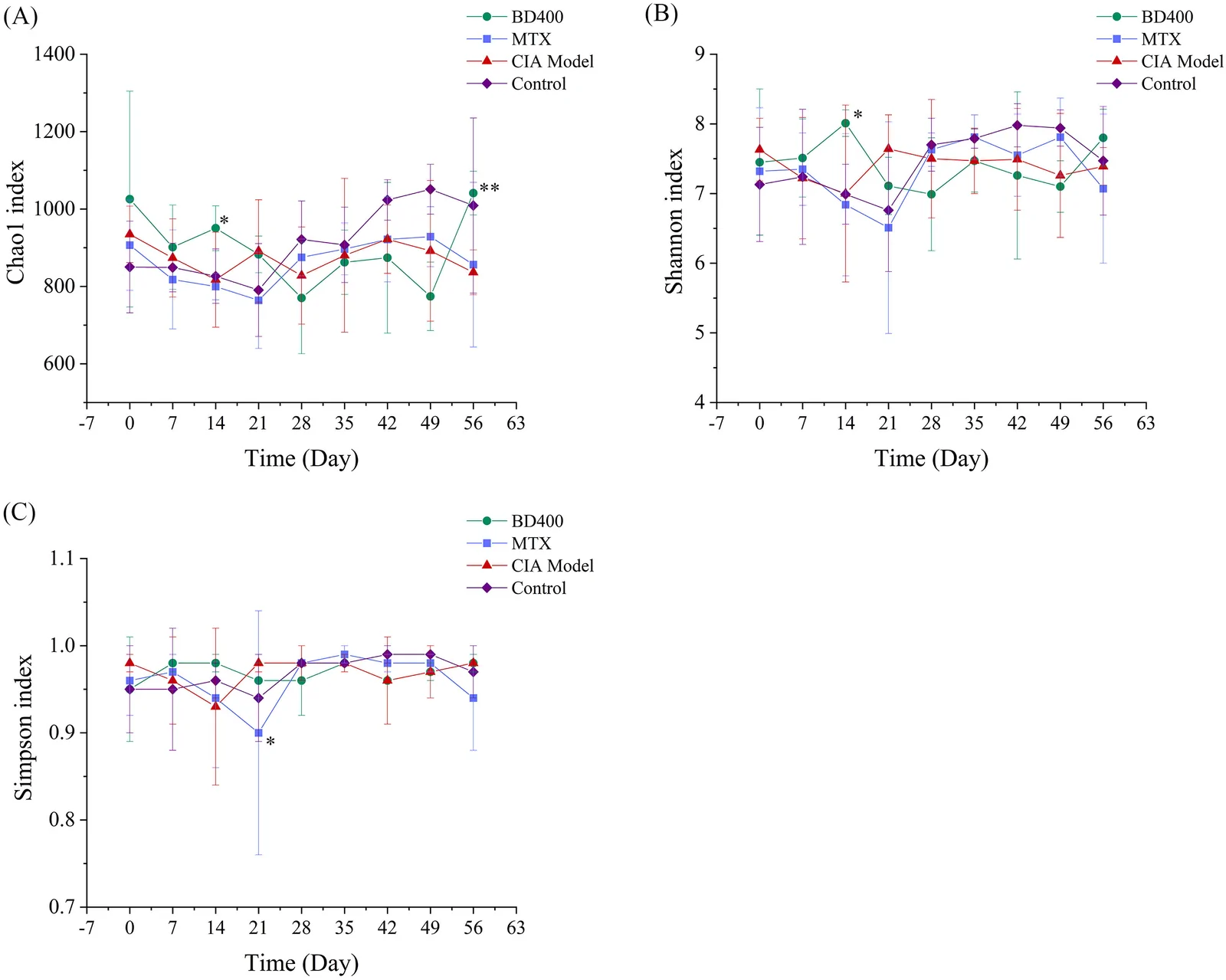
Longitudinal changes of gut microbial α-diversity indices within each treatment group. **(A)** Chao1 index (t = 14, *P* = 0.03; t = 56, *P* = 0.03, Wilcoxon); **(B)** Shannon index (t = 14, *P* = 0.03, Wilcoxon); **(C)** Simpson index (t = 21, *P* = 0.01, Wilcoxon). *P < 0.05; **P < 0.01 vs CIA model group at the same time point.

### 3.3 Longitudinal analysis of gut microbial β diversity

To visualize the temporal shifts of each group relative to its own baseline, we generated a PCoA plot based on the Jaccard distance incorporating all experimental groups and all sampling time-points (**Figure 4**). Sample distribution exhibited apparent temporal shifts along the PCoA1 axis. Although partial overlap was observed among experimental groups, 95% confidence ellipses indicated community separation between groups, suggesting that both time and treatment shaped gut microbial profiles. Time-series principal coordinate analysis (PCoA) was performed based on two complementary β-diversity metrics: Jaccard distance (**Supplementary Figures 3A–I**) and Bray-Curtis distance (**Supplementary Figures 3J–R**), to track longitudinal shifts in gut microbial community structure across control, CIA model, MTX and BD400 groups from Day 0 to Day 56. Quantitative statistical verification of community dissimilarity was further conducted *via* ANOSIM and Adonis tests using weighted-UniFrac distance at each time point (**Table 1**).

**Figure 4:**
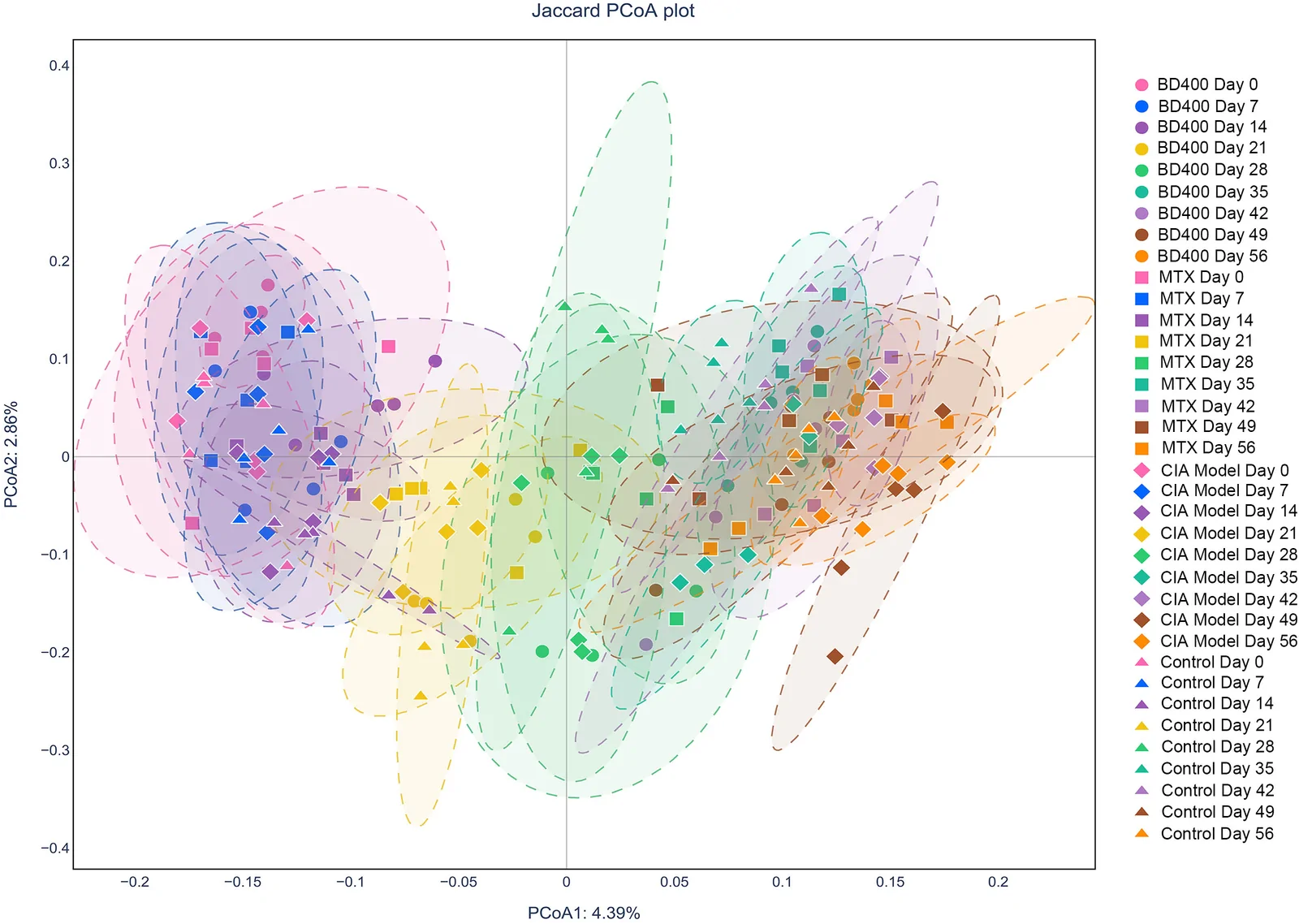
Principal-coordinates analysis (PCoA) of gut microbiota based on Jaccard distance. Each dot represents an individual rat sample. Points are colored according to sampling time, and shaped by experimental group. Ellipses denote 95% confidence intervals for each experimental group. PCoA1 and PCoA2 indicate the percentage of total variance explained by the corresponding axes.

**Table 1:** β-diversity statistics based on weighted-UniFrac distance at each experimental time-point.

Time (Day)	ANOSIM *R* statistic	Adonis *R^2^*	Adonis *F* model	ANOSIM *P* value	Adonis *P* value	Significant
0	0.033	0.197	1.312	0.286	0.203	ns
7	-0.044	0.167	1.066	0.677	0.388	ns
14	0.144	0.270	1.971	0.038	0.021	*
21	0.299	0.420	3.867	0.004	0.002	**
28	0.078	0.195	1.292	0.120	0.169	ns
35	0.218	0.290	2.174	0.004	0.002	**
42	0.073	0.192	1.269	0.145	0.180	ns
49	0.189	0.256	1.836	0.009	0.007	**
56	0.253	0.279	2.059	0.003	0.025	**

ns, not significant. *P < 0.05, **P < 0.01.

At baseline (Day 0 and Day 7), both PCoA ordinations showed extensive overlap between control and CIA model clusters. Consistently, **Table 1** revealed non-significant inter-group differences at Day 0 (ANOSIM *R* = 0.033, *P* = 0.286; Adonis *R²* = 0.197, *P* = 0.203) and Day 7 (ANOSIM *R* = –0.044, *P* = 0.677; Adonis *R²* = 0.167, *P* = 0.388), indicating highly homogeneous initial gut microbiota before obvious CIA pathological damage. Starting from Day 14, spatial separation between control and CIA model groups emerged in PCoA plots. The weighted-UniFrac statistics confirmed significant community divergence at Day 14 (*P* < 0.05), which became highly significant at Day 21, 35, 49 and 56 (*P* < 0.01). The ANOSIM *R* values rose continuously from 0.144 (Day 14) to 0.299 (Day 21), demonstrating gradually enlarging dissimilarity between healthy and CIA-disrupted gut microbiota as arthritis progressed. Adonis *R²* values also peaked at Day 21 (0.420), showing that grouping factor explained the largest proportion of microbial variance at this severe disease stage. These results jointly proved that CIA modeling induced progressive, statistically robust remodeling of gut bacterial communities that deviated far from the healthy control microbial profile.

Compared with the CIA model group, MTX intervention aggravated gut microbial structural dysbiosis. At all significant time points (Day 14, 21, 35, 49, 56), the MTX cluster exhibited larger spatial displacement away from the control ellipse than the CIA model group in both Jaccard and Bray-Curtis PCoA. At Day 21 and Day 42, the MTX ellipse shared minimal overlap with the CIA model ellipse, revealing a distinct dysbiotic microbial signature unique to MTX treatment. Statistical data in **Table 1** supported this observation: the maximum inter-group dissimilarity (ANOSIM *R* = 0.299 at Day 21) corresponded to the most severe community segregation involving the MTX group. MTX reshaped the intestinal microbiome into a configuration distinguishable from the intrinsic microbiota disturbance triggered by CIA alone, introducing extra structural perturbation on the basis of CIA-induced dysbiosis.

Across all serial sampling days and both distance-based PCoA algorithms, the BD400 group maintained persistent, extensive overlap with the healthy control cluster, with markedly smaller ordination separation from control compared with CIA model and MTX groups. Unlike MTX, BD400 supplementation prevented drastic deviation from the healthy microbial baseline; at late intervention stages (Day 42–56), the BD400 ellipse converged closer to the control cluster, while CIA model and MTX groups remained distinctly segregated from healthy microbiota. Consistent with the PCoA visualization, the weighted-UniFrac ANOSIM and Adonis statistics illustrated that the strong inter-group dissimilarity detected at Day 14, 21, 35, 49 and 56 was primarily driven by the divergent microbiota of CIA model and MTX groups, whereas BD400-treated mice retained a community composition analogous to that of healthy controls. Collectively, results from Jaccard, Bray-Curtis and weighted-UniFrac distances mutually validated that BD400 effectively restrained CIA-induced gut community structural shift and preserved normal intestinal microbiota architecture, whereas MTX monotherapy failed to recover microbial homeostasis and exacerbated community dissimilarity relative to healthy rats.

### 3.4 Analysis of gut microbiota composition and dominant genera

To further identify which species are responsible for these differences, we conducted a species composition analysis (**Figure 5**). At baseline Day 0, the control group and CIA model group shared highly consistent taxonomic profiles: Firmicutes dominated the phylum level, accompanied by minor proportions of Campylobacterota, Verrucomicrobiota and Desulfobacterota; the core dominant genera included *Ligilactobacillus*, *Ruminococcus* and *Lachnospiraceae*_NK4A136_group. After CIA induction, obvious taxonomic divergence emerged between the two groups. In the CIA Model group (**Figure 5B**), the relative abundance of beneficial *Ligilactobacillus* continuously declined over time, while the proportions of conditional pathogenic genera such as *Helicobacter* and *Desulfovibrio* rose markedly at late stages (Day 42–56). In contrast, the healthy control group (**Figure 5A**) maintained stable high abundance of *Ligilactobacillus* and *Lactobacillus* throughout the whole period, with only low, steady levels of opportunistic bacteria. At Day 56, the control group retained a large proportion of *Ligilactobacillus*, whereas this genus became one of the minor taxa in CIA model mice. These findings indicated that CIA modeling disrupted the balanced composition of core commensal bacteria, suppressed probiotic-like genera, and enriched potential pro-inflammatory intestinal microbes.

**Figure 5:**
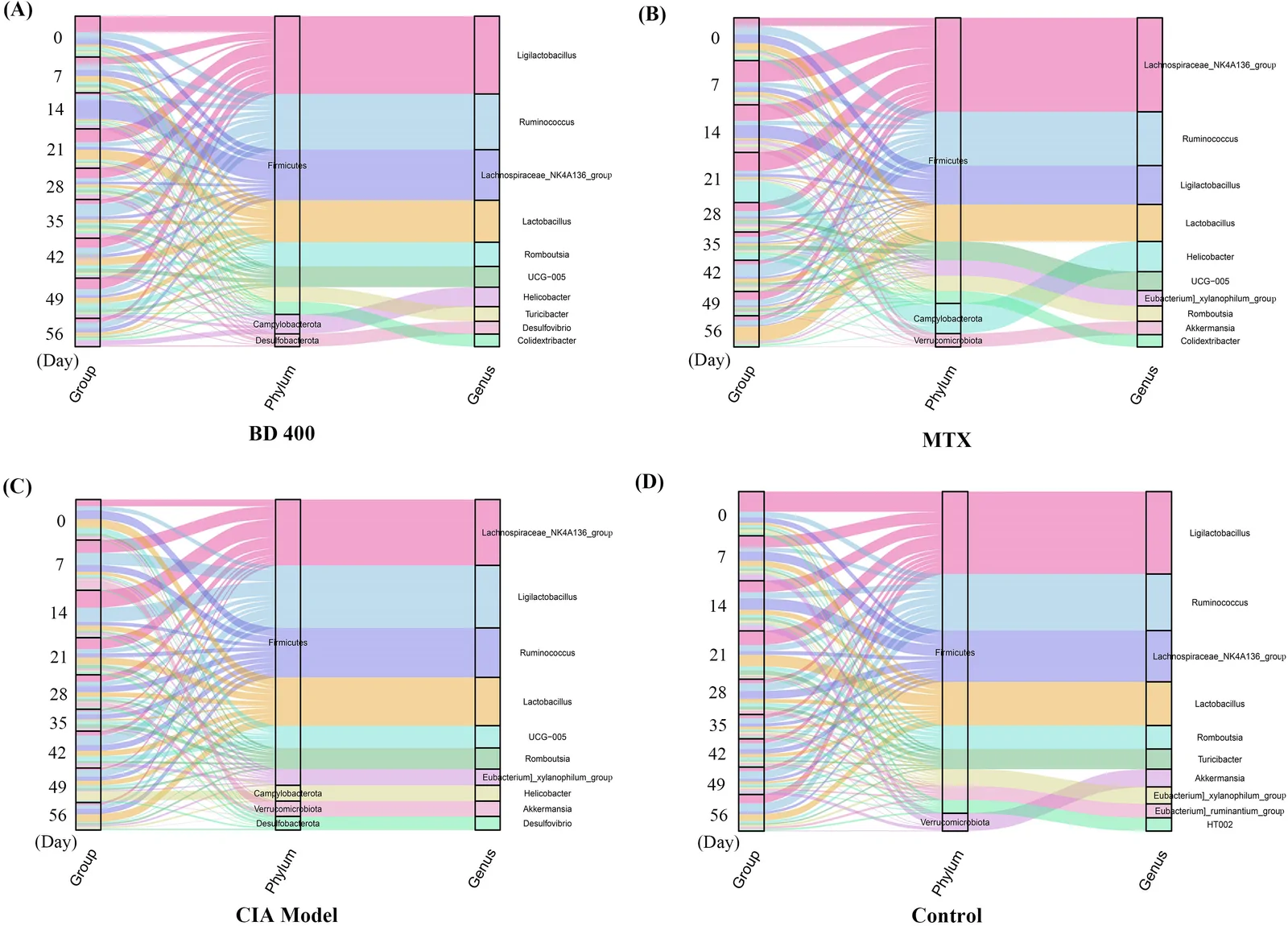
Sankey plots illustrating longitudinal shifts in gut microbial composition at phylum and genus levels. **(A)** Control group; **(B)** CIA model group; **(C)** MTX group; **(D)** BD400 group.

Compared with the CIA model group, MTX intervention induced more severe disturbance of gut microbial taxonomic structure (**Figure 5C**). At the phylum level, the MTX group exhibited larger fluctuations in the ratio of Firmicutes to minor phyla than the CIA model group. At the genus level, MTX treatment caused a drastic depletion of *Ligilactobacillus* starting at Day 14; meanwhile, the relative abundance of *Helicobacter* increased far more drastically than that in the CIA model group, and unique enrichment of *Eubacterium*_*xylanophilum*_group was observed at late time points. Notably, the dominant genus order of the MTX group was completely rearranged relative to the CIA model group after Day 21. The MTX group failed to restore the high abundance of *Ligilactobacillus* characteristic of healthy rats, and instead aggravated the outgrowth of pro-inflammatory taxa on the basis of CIA-induced flora imbalance.

Across all sampling time points, the taxonomic succession pattern of the BD400 group (**Figure 5D**) was highly consistent with the healthy control group, and significantly more stable than both CIA model and MTX groups. At the phylum level, BD400 supplementation sustained a stable Firmicutes-dominated profile without abnormal expansion of minor phyla. At the genus level, BD400 mice maintained persistently high relative abundance of *Ligilactobacillus* and *Lactobacillus* from Day 0 to Day 56; the abundance of *Helicobacter* and *Desulfovibrio* remained at low levels, with no late-stage enrichment observed in CIA model and MTX groups. Unlike MTX, BD400 effectively prevented the CIA-mediated depletion of beneficial *Lactobacilli* and restrained the proliferation of potential pathogenic genera. At the end of the experiment (Day 56), the core genus composition of BD400-treated rats closely resembled that of control rats, while CIA model and MTX groups displayed drastically altered genus profiles. Collectively, BD400 supplementation may be linked to relatively consistent longitudinal taxonomic structure of gut microbiota at both phylum and genus levels, which could favor retention of core beneficial gut-microbiota signatures resembling healthy controls under CIA pathological conditions.

### 3.5 Changes in gut microbiota composition at genus level

We further analyzed the temporal changes of the different genera as mentioned in Section 3.4. For the *Lactobacillaceae*-related genera (including *Ligilactobacillus*, *Lactobacillus*, *Bifidobacterium*) (in **Figures 6A, D, L**), at baseline (Day 0), control and BD400 groups possessed higher *Ligilactobacillus* abundance, whereas CIA and MTX groups started at low levels. The control group showed a steady decline until Day 35 with a mild late rebound; the CIA group fluctuated sharply with dual peaks at Day 14 and 21; the MTX group gradually decreased after Day 14 and remained the lowest in late stages; and the BD400 group maintained consistently higher abundance than the CIA and MTX groups throughout the trial. For *Lactobacillus*, the control group peaked sharply at Day 14 and then declined, while the CIA and MTX groups shared a Day 14 trough followed by a synchronized Day 21 peak; the MTX group stayed at the lowest level from Day 28 onward, and the BD400 group fluctuated moderately above the MTX group. As for *Bifidobacterium*, all groups had comparable starting values; the CIA group generated the highest peak at Day 21, the BD400 group showed moderate oscillations with a minor secondary peak at Day 42, and the control and MTX groups sustained low, gentle fluctuations, with the MTX group reaching the minimum at Day 56.

**Figure 6:**
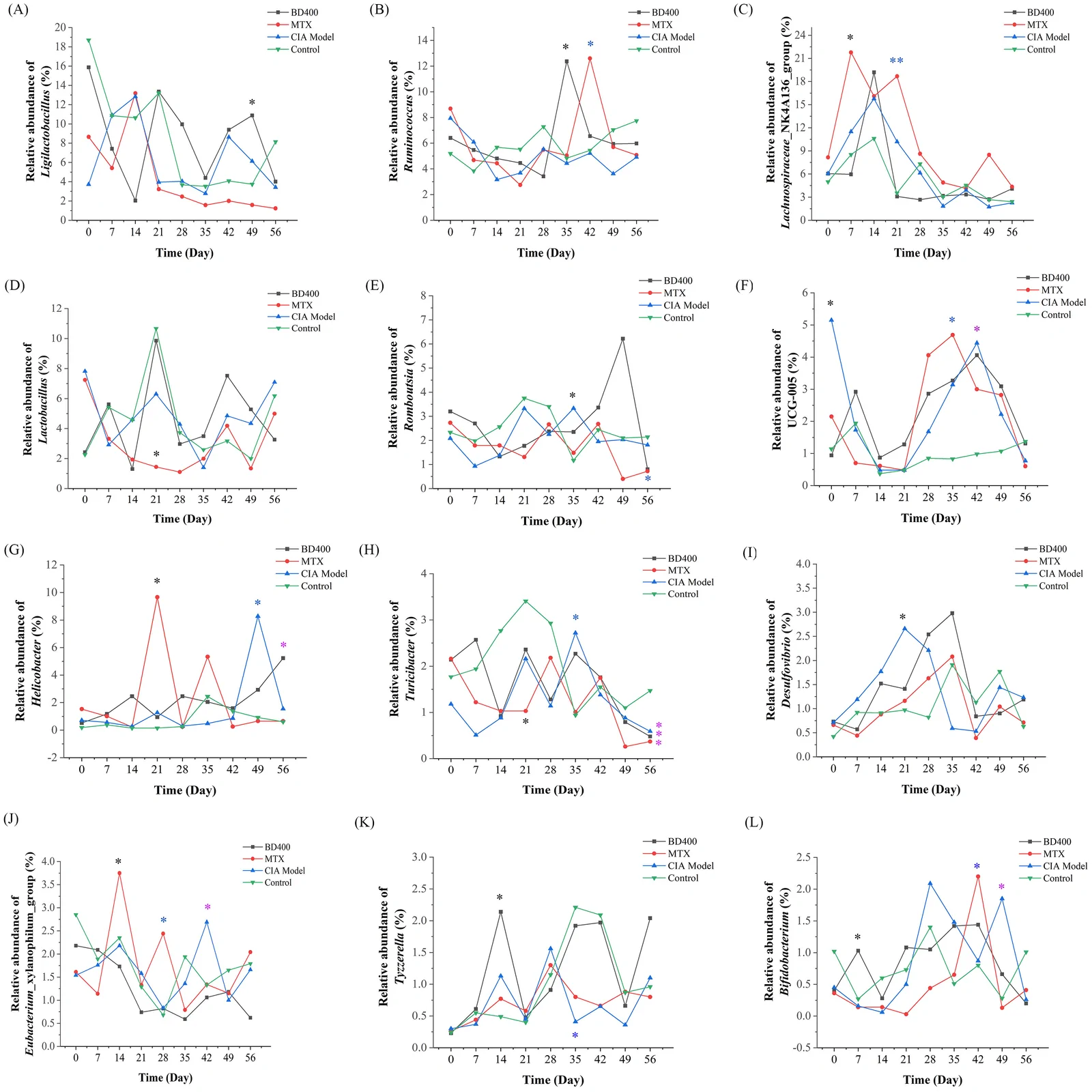
The changes in the gut microbiota of each group in rats over time at the genus level. Different colored asterisks indicate the significant differences at different time points. *P < 0.05, **P < 0.01 against the control group. The different colors of * indicate that they are at different time points.

The fixed effect test showed that among the three *Lactobacillaceae*-related genera, only *Lactobacillus* had a significant main effect over time (from Day 0 to Day 56, *P* < 0.01), suggesting that intervention duration may contribute to the temporal dynamics of *Lactobacillus* abundance. Pairwise LSD comparisons of estimated marginal means revealed obvious temporal variation in *Lactobacillus* relative abundance across Day 7 to Day 56. The abundance of *Lactobacillus* peaked significantly at Day 21 and Day 35 relative to Day 14 (*P* < 0.05). By Day 56, *Lactobacillus* levels decreased sharply and were significantly lower than Day 14, 21, 28, 35, 49 and Day 7 (*P* < 0.05). No significant differences were detected for Day 42 against all other time points (*P* > 0.05). Collectively, *Lactobacillus* abundance presented an increasing-then-decreasing dynamic profile over the experimental timeline, with its minimum value observed at Day 56. LSD pairwise comparisons of estimated marginal means identified limited temporal variation in *Ligilactobacillus* relative abundance from Day 7 to Day 56. Only Day 35 exhibited a significantly higher abundance of *Ligilactobacillus* relative to Day 14 (mean difference = 6.605, *P* = 0.021). The comparison between Day 35 and baseline Day 7 showed a borderline significant increasing trend (*P* = 0.051). All other pairwise comparisons across all time points failed to reach statistical significance (*P* > 0.05). Overall, *Ligilactobacillus* abundance remained largely consistent over the experimental timeline, with its peak value detected at Day 35. LSD pairwise comparisons of estimated marginal means revealed significant temporal shifts in *Bifidobacterium* relative abundance from Day 7 to Day 56. Compared with Day 14, *Bifidobacterium* abundance was significantly reduced at Day 28 (*P* = 0.028), Day 35 (*P* = 0.019) and Day 42 (*P* = 0.018). Relative to baseline Day 7, both Day 35 (*P* = 0.039) and Day 42 (*P* = 0.036) showed significantly elevated *Bifidobacterium* levels. All other pairwise contrasts yielded non-significant differences (*P* > 0.05). Collectively, *Bifidobacterium* abundance increased gradually and reached significantly higher levels at the late-middle experimental stage (Day 35–42) relative to the early baseline and Day 14.

For short-chain fatty acid-producing Lachnospiraceae (including *Ruminococcus*, *Lachnospiraceae*_NK4A136_group, *Eubacterium*_*xylanophilum*_group) (in **Figures 6B, C, J**), all three genera shared a universal Day 14 or Day 21 peak trend across groups. *Ruminococcus* reached a shared maximum at Day 21 for the CIA and MTX groups; the BD400 group’s Day 21 peak was the highest among all groups, and the MTX group dropped continuously afterward. For *Lachnospiraceae*_NK4A136_group, every group peaked synchronously at Day 14 before a general fall until Day 35; MTX group maintained the lowest abundance in the late phase. *Eubacterium*_*xylanophilum*_group peaked earliest at Day 14 in the MTX group; the CIA group rose to a moderate peak at Day 21 and then slowly declined, while the control group kept flat, low levels with tiny fluctuations.

The fixed effect test showed that in the short-chain fatty acid-producing Lachnospiraceae, *Ruminococcus* had a significant main effect in Time (*P* < 0.05) and Group×Time (*P* < 0.01), *Lachnospiraceae*_NK4A136_group had a significant main effect in Group (*P* < 0.001) and Time (*P* < 0.001), and *Eubacterium*_*xylanophilum*_group had a significant main effect in Time (*P* < 0.01). LSD pairwise comparisons of estimated marginal means revealed prominent temporal fluctuations in *Ruminococcus* abundance. Day 42 represented the nadir of *Ruminococcus*, with significantly lower abundance than Day 7, 14, 21 and 28 (*P* < 0.05). Day 35 also had reduced *Ruminococcus* levels relative to Day 14 and Day 21 (*P* < 0.05). No significant inter-timepoint differences were detected at Day 49 and Day 56 against all other sampling days (*P* > 0.05). Overall, *Ruminococcus* abundance declined sharply to a significant minimum at Day 42, with no obvious variation in the late experimental stage (Day 49–56). LSD pairwise comparisons revealed dual layers of variation in *Lachnospiraceae*_NK4A136_group. At the group level, MTX treatment yielded significantly higher abundance than BD400, CIA model and Control (*P* < 0.05), with no differences among the three non-MTX groups. Over time, *Lachnospiraceae*_NK4A136_group abundance was significantly higher at Day 21–56 versus Day 14 (*P* < 0.05), with no significant difference between Day 7 and Day 14 (*P* = 0.088). Relative to Day 21, its abundance increased markedly at Day 35–56 (*P* < 0.05). Day 7 exhibited lower levels than Day 28–56 (*P* < 0.05). This taxon presented a sustained upward trend throughout the experiment. LSD pairwise tests (df = 112) revealed that *Eubacterium*_*xylanophilum*_group abundance was significantly higher at Day 21–56 versus Day 14 (*P* = 0.001–0.014), and Day 14 also had lower levels than Day 7 (*P* = 0.031). No significant differences were detected between Day 7 and Day 21–56 (*P* > 0.05), nor among Day 21, 28, 35, 42, 49 and 56 (*P* > 0.05). This taxon rose sharply after Day 14 and remained stable thereafter.

For other commensal Firmicutes (including *Romboutsia*, *Turicibacter*, *Desulfovibrio*, *Tyzzerella*) (in **Figures 6E, H, I, K**), baseline levels of these four genera were all similar and low across groups. *Romboutsia* only formed a prominent single peak in the BD400 group at Day 42, with the other three groups staying within narrow medium-low fluctuation ranges. *Turicibacter* was most abundant in the control group, with a broad peak from Day 14 to 21; The MTX group trended downwards after Day 21 and hit the lowest value on Day 56. The *Desulfovibrio* levels of all groups climbed together to a Day 21 peak, after which the BD400 group retained medium-high abundance while the MTX group fell to the bottom at the end. For *Tyzzerella*, the BD400 group peaked first at Day 14, the control and CIA groups peaked at Day 28, and the MTX group gradually declined after Day 28 and remained the lowest late in the experiment.

The fixed effect test showed that in the other commensal Firmicutes category, *Turicibacter* had significant main effects in Group (*P* < 0.001) and Time (*P* < 0.001), *Desulfovibrio* had a significant main effect in Time (*P* < 0.01), and *Tyzzerella* had significant main effects in Group (*P* < 0.05), Time (*P* < 0.001), and Group×Time (*P* < 0.01). LSD pairwise tests (df = 112) identified significant temporal differences of *Romboutsia* only between Day 56 and four earlier time points. Day 56 exhibited markedly higher abundance than Day 21 (mean difference = 1.171, *P* = 0.037), Day 28 (1.300, *P* = 0.021), Day 42 (1.240, *P* = 0.027) and Day 49 (1.322, *P* = 0.019). No other pairwise comparisons across Day 7–56 reached statistical significance (*P* > 0.05). LSD pairwise comparisons were conducted for group (df = 15) and time (df = 112) effects on *Turicibacter*. For group comparisons: Control exhibited markedly higher abundance than BD400 (*P* = 0.004), CIA model (*P* = 0.001) and MTX (*P* < 0.001). BD400 was enriched relative to MTX (*P* = 0.027), while CIA Model had no significant difference *vs* BD400 or MTX. For temporal comparisons: Day 21 had the highest *Turicibacter* level, which was significantly higher than Day 14, 42, 49, 56 and Day 7 (*P* = 0.007, 0.030, < 0.001, < 0.001, 0.022, respectively). Day 49 and Day 56 showed significantly lower abundance versus Day 21, 28, 35, 42, and baseline Day 7 (*P* < 0.05). All other pairwise time contrasts were non-significant. LSD temporal pairwise tests (df = 112) revealed significant shifts of *Desulfovibrio*. Day 42 showed higher abundance than Day 21 (*P* = 0.029), Day 28 (*P* = 0.005) and Day 35 (*P* = 0.002). Day 56 was lower than Day 28 (*P* = 0.022) and Day 35 (*P* = 0.012). Baseline Day 7 exhibited higher levels than Day 21 (*P* = 0.042), Day 28 (*P* = 0.007) and Day 35 (*P* = 0.004). No other pairwise contrasts reached *P* < 0.05. LSD pairwise tests were performed to assess group (df = 15) and temporal (df = 112) changes in *Tyzzerella*. For group effects: BD400 possessed significantly higher *Tyzzerella* abundance than CIA Model (*P* = 0.015) and MTX (*P* = 0.013), with no other intergroup differences observed. For time effects: Day 7 and Day 42 showed the highest *Tyzzerella* levels. Day 21 displayed significantly lower abundance versus Day 14, 28, 35, 42 and 56 (*P* < 0.05). Day 49 was markedly reduced compared with Day 28, 35 and 42 (*P* < 0.05). Baseline Day 7 was significantly enriched relative to Day 14, 28, 35, 42 and 56 (*P* < 0.05). All other temporal pairwise contrasts were non-significant.

For Proteobacteria and miscellaneous taxa (including UCG-005, *Helicobacter*) (in **Figures 6F, G**), UCG-005 was highest in CIA group at Day 14 before a steep drop; both the CIA and MTX groups then rebounded to a major peak at Day 35; the control group maintained persistently low values, and the BD400 group fluctuated between high and low groups. *Helicobacter* was nearly undetectable at Day 0; the MTX group exhibited a sharp Day 14 spike (the highest reading of all time points), the CIA group slowly accumulated to a secondary peak at Day 49, and the control group kept negligible abundance throughout, with the BD400 group remaining far lower than the MTX and CIA groups after Day 14.

The fixed effect test showed that among Proteobacteria and miscellaneous taxa, only UCG-005 had a significant main effect in Group (*P* < 0.05) and Time (*P* < 0.001). LSD pairwise comparisons based on estimated marginal means were conducted to evaluate intergroup and temporal alterations of UCG-005. For group comparisons (df = 15): Only BD400 showed significantly higher UCG-005 abundance relative to Control (*P* = 0.005), while no other intergroup differences were observed. For time-point comparisons (df = 112): Day 56 presented the highest UCG-005 levels, which were significantly higher than Day 28, Day 35, Day 42 and Day 49 (*P* < 0.05). Day 14 and Day 21 exhibited markedly lower abundance versus Day 28, Day 35, Day 42 and Day 49 (*P* < 0.01). Day 42 was significantly enriched relative to baseline Day 7 (*P* = 0.035), whereas Day 14 was significantly depleted compared with Day 7 (*P* = 0.044). All other temporal pairwise contrasts were non-significant.

To quantitatively characterize the longitudinal dynamic shifts of core differential commensal genera across the control, CIA model, MTX and BD400 groups, pairwise time-series differential abundance analysis was performed with FDR-adjusted *q*-values to identify statistically significant temporal fluctuations (**Supplementary Table 1**). Line graphs further visualized the continuous relative abundance trajectories of nine key genera over the 56-day experimental period (**Figure 7**).

**Figure 7:**
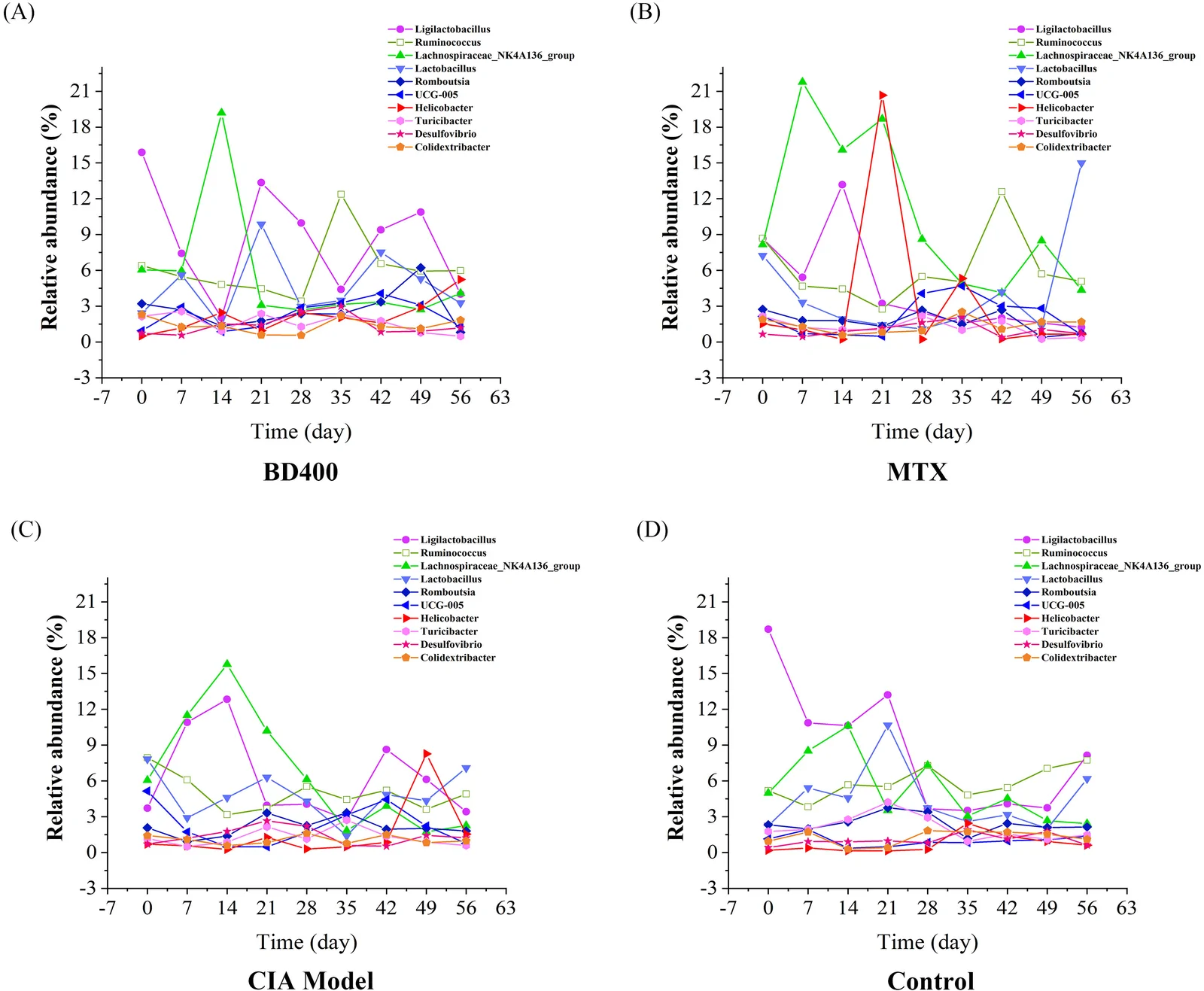
Longitudinal dynamic changes in the relative abundance of key gut genera. **(A)** BD400 group; **(B)** MTX group; **(C)** CIA model group; **(D)** Control group.

In the BD400 group (**Figure 7A**), consistent with FDR-corrected pairwise comparisons, the gut microbiota exhibited early, sustained and statistically significant upregulation of lactic acid-producing and SCFA-synthesizing genera starting at Day 7 (*q* < 0.05). For *Ligilactobacillus* and *Lactobacillus*, marked elevation was detected at Day 0 *vs* Day 7 (*q* < 0.05), followed by recurrent significant enrichment at Day 21, Day 49 and Day 56 relative to preceding timepoints, reflected by persistently higher relative abundance throughout the trial. *Lachnospiraceae*_NK4A136_group showed a sharp peak at Day 14 with significant temporal upregulation (*q* < 0.05); its abundance remained markedly higher than baseline until Day 42. For *Ruminococcus*, *Romboutsia*, UCG-005, *Colidextribacter*, significant positive fold changes were observed at Day 7 and Day 14 (*q* < 0.05), maintaining stable elevated abundance for the entire intervention window. In contrast, *Helicobacter* and *Desulfovibrio* displayed consistent significant depletion across most consecutive time comparisons (*q* < 0.05), with their relative abundance suppressed to low levels from Day 14 onward.

In the MTX group (**Figure 7B**), pairwise FDR screening demonstrated that MTX treatment aggravated CIA-driven gut dysbiosis, producing the broadest range of significantly depleted beneficial genera across all time intervals. For *Ligilactobacillus*, *Lactobacillus*, *Lachnospiraceae*_NK4A136_group and *Romboutsia*, significant negative log_2_FC values were identified from Day 14 to Day 56 (*q* < 0.05). Although a transient abundance peak of *Lachnospiraceae*_NK4A136_group was observed at Day 14 in the line chart, this elevation was not statistically sustained, followed by prolonged significant depletion. *Helicobacter* and *Desulfovibrio* exhibited consistent significant enrichment across mid-to-late time points (*q* < 0.05), with their relative abundance maintained at higher levels than in both the BD400 and control groups. Only minor, non-sustained significant shifts were detected for *Colidextribacter* and UCG-005, without the persistent enrichment seen in the BD400 group.

In the CIA model group (**Figure 7C**), FDR-adjusted *q*-values confirmed that CIA induction triggered delayed, irregular, time-dependent microbial dysregulation without early protective genus enrichment, and most significant taxonomic shifts emerged after the booster immunization (Day 21). For *Ligilactobacillus* and *Lactobacillus*, no significant upregulation was observed at Day 7 (*q* > 0.05). Transient weak elevation appeared at Day 14, followed by sustained significant depletion from Day 21 to Day 42 (*q* < 0.05), matching the continuous decline in relative abundance shown in the trajectory plot. For *Lachnospiraceae*_NK4A136_group, *Ruminococcus*, *Romboutsia*, significant temporal depletion was detected at Day 21, Day 35 and Day 42 (*q* < 0.05). Their relative abundance peaked only briefly at Day 14 before progressive suppression for the remainder of the experiment. For Desulfovibrio, Helicobacter and Turicibacter, multiple consecutive pairwise comparisons revealed significant enrichment starting at Day 28 (*q* < 0.05), with their relative abundance gradually rising at late experimental stages.

In the control group (**Figure 7D**), FDR-adjusted pairwise comparisons indicated minimal, transient microbial fluctuations in the control group, with far fewer statistically significant temporal genus shifts relative to the three treatment groups. *Ligilactobacillus* showed an initial high abundance at Day 0 but underwent continuous significant depletion at Day 7 and Day 14 (*q* < 0.05), with no subsequent recovery to baseline levels for the full trial duration. SCFA-producing genera only exhibited isolated, short-term significant changes at discrete time points (e.g., *Lachnospiraceae*_NK4A136_group at Day 42, *q* < 0.05), without stable long-term upregulation. No consistent significant depletion of *Helicobacter* and *Desulfovibrio* was detected at any consecutive time comparison (*q* > 0.05), corresponding to the moderate, inconsistent abundance fluctuations visualized in the trajectory curves.

Statistical FDR testing paired with genus-level abundance trajectories revealed divergent temporal gut microbial remodeling profiles across experimental groups. Only BD400 gavage induced early (Day 7) and sustained significant enrichment of lactic acid bacteria and butyrate-producing commensals, alongside persistent suppression of pro-inflammatory pathobionts. By contrast, CIA modeling and MTX treatment both caused progressive, statistically significant depletion of beneficial core genera and enrichment of harmful taxa, while the control group displayed limited, unsustained microbial variation without targeted regulation of functional gut commensals.

### 3.6 LEfSe-based identification of key differential gut microbiota across experimental groups

Species composition analysis examines the differences in the abundance of the dominant species among different groups. To avoid overlooking the roles played by low-abundance species during the development and progression of arthritis, we employed the LEfSe method to analyze the significant differences in species among different groups. **Figure 8** comprehensively presents the species with significant differences among these groups at the phylum, class, order, family, genus, species levels at each time point, as well as their evolutionary relationships. At baseline (Day 0), LEfSe cladogram analysis revealed no phylum-level differential taxa across the four experimental cohorts (**Figure 8A**), indicating consistent initial gut microbial composition prior to intervention and immunization. Significant gut microbial remodeling was first detected in the BD400 group as early as Day 7 following continuous oral gavage. Phylogenetic biomarker screening *via* LEfSe evolutionary trees identified *Turicibacter* and *Bifidobacterium* as the genera with significantly altered relative abundance in the BD400 group (**Figure 8B**).

**Figure 8:**
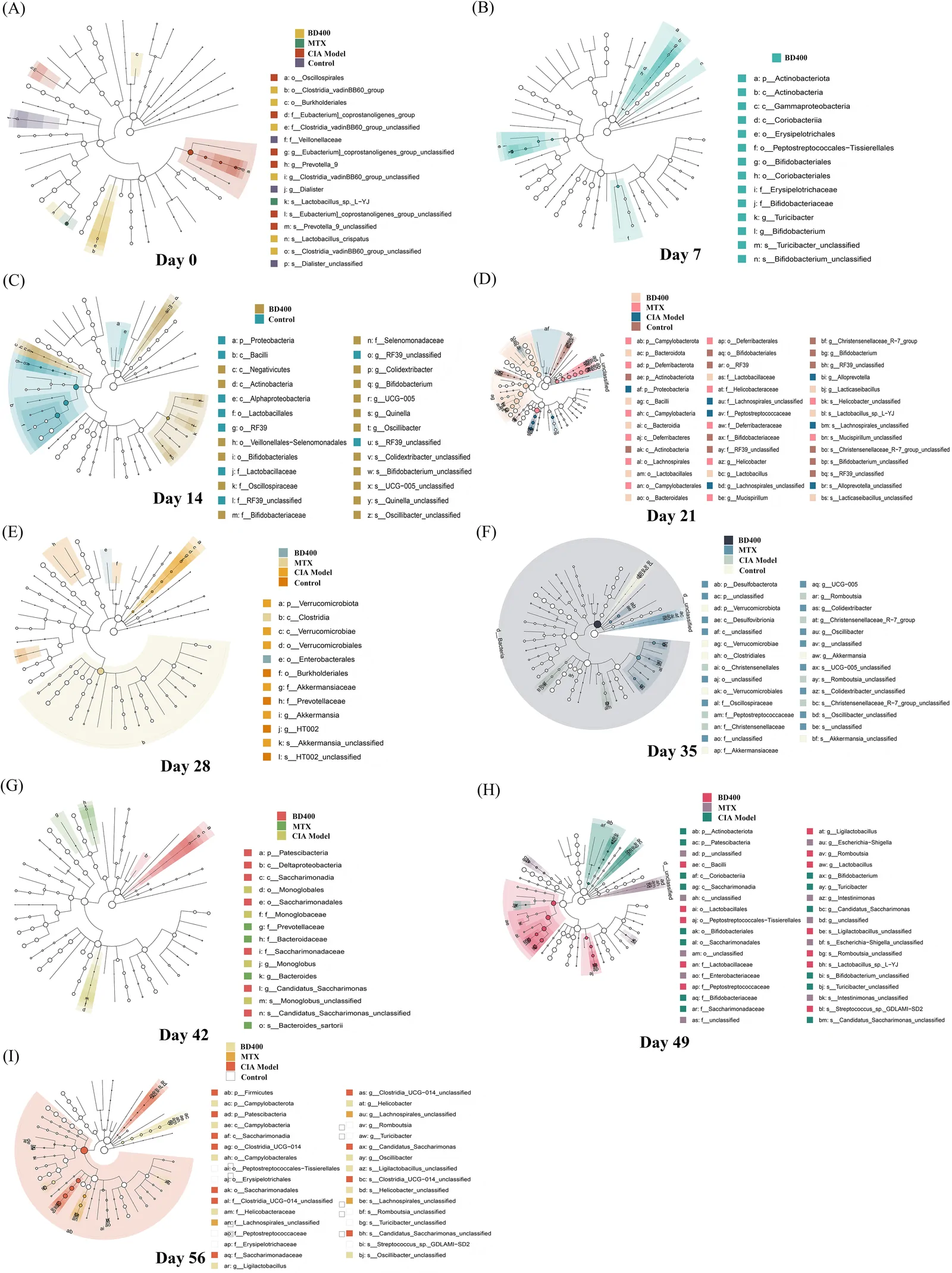
LEfSe cladogram illustrating the evolutionary relationships of groups with differential taxa at sequential sampling time-points. Note that groups without labeling indicate no differential taxa were identified at the corresponding time point.

By Day 14, temporal microbiota divergence further expanded: the control group exhibited prominent shifts within the phylum Proteobacteria, whereas five genera (*Colidextribacter*, *Bifidobacterium*, UCG-005, *Quinella*, *Oscillibacter*) were characterized as differential biomarkers exclusive to the BD400 group (**Figure 8C**). At Day 21, coinciding with booster immunization shown in **Figure 1**, the CIA model group developed two signature taxa, namely *Lachnospirales*_unclassified and *Alloprevotella* (**Figure 8D**). Distinct from CIA-driven microbial perturbation, BD400 intervention induced sustained differential enrichment of *Lactobacillus* and *Lacticaseibacillus*, with consistent statistical differences retained through Day 49 and Day 56 (**Figures 8H, I**). Overall, the taxonomic shifts triggered by BD400 displayed remarkable temporal stability throughout the trial.

In contrast, CIA modeling induced progressive, multi-layered gut dysbiosis with continuously renewed differential genera over the post-immunization period: Day 28 featured altered *Akkermansia* (**Figure 8E**); Day 35 distinguished *Romboutsia* and *Christensenellaceae*_R-7_group (**Figure 8F**); Day 42 identified *Monoglobus* as a CIA-specific biomarker (**Figure 8G**); Day 49 saw significant fluctuations in *Bifidobacterium* and *Turicibacter* alongside *Candidatus*_*Saccharimonas* (**Figure 8H**); and the endpoint Day 56 exhibited differential *Clostridia*_UCG-014_unclassified (**Figure 8I**). Such widespread microbial disturbance induced by CIA spanned five major phyla: Firmicutes, Actinobacteriota, Verrucomicrobiota, Patescibacteria and Proteobacteria. Of note, the control group only presented sporadic phylum-level fluctuations at discrete time points, including Proteobacteria (Day 14), Verrucomicrobiota (Day 21) and Actinobacteriota (Day 35), without broad-spectrum dysbiosis.

Notably, the taxonomic signature of the MTX treatment cohort was phylogenetically segregated from the remaining three groups, with differential biomarkers exclusively derived from Campylobacterota, Deferribacterota and Desulfobacterota—phyla unaffected in control, CIA or BD400 groups at any sampling timepoint.

For the BD400 group, the gut microbiome underwent sequential, ordered remodeling starting from Day 7 post-administration: Day 7: *Turicibacter* and *Bifidobacterium*; Day 14: *Colidextribacter*, *Bifidobacterium*, UCG-005, *Quinella*, *Oscillibacter*; Day 21: *Lactobacillus*, *Lacticaseibacillus*; Day 28: Enterobacterales. The microbial community of BD400-treated rats reached a relatively steady state at Day 35, yet still maintained characteristic genus-level shifts at late time points: Day 49 (*Ligilactobacillus*, *Romboutsia*, *Lactobacillus*) and Day 56 (*Ligilactobacillus*, *Helicobacter*, *Oscillibacter)*.

## 4 Discussion

Based on our previous research on the prevention of arthritis with BD400, this study further demonstrated the dynamic changes in the gut microbiota of CIA rats as arthritis progressed. *B. animalis* BD400 can affect the abundances of *Clostridia*_vadinBB60_group_unclassified, *Turicibacter*, *Bifidobacterium*, *Colidextribacter*, UCG-005, *Quinella*, *Oscillibacter*, *Lactobacillus* and *Lacticaseibacillus* in the early stage of RA, and the abundances of *Candidatus*_*Saccharimonas*, *Ligilactobacillus*, *Romboutsia*, *Lactobacillus*, *Helicobacter* and *Oscillibacter* in the later stage of CIA. Ultimately, intervention with *B. animalis* BD400 may exert protective effects against arthritis in the CIA model.

Mounting evidence has corroborated the critical role of gut microbiota in the initiation and progression of RA. Gut dysbiosis can disrupt intestinal barrier integrity, trigger aberrant mucosal immune activation, and promote the translocation of microbial components into circulation, which further drives systemic autoimmunity and joint inflammation. Many studies have shown that at this preclinical stage of RA, the relative abundance of *Bacteroidaceae* is decreased, while that of *Ruminococcaceae*, *Lachnospiraceae*, and *Desulfovibrinocaceae* is increased (13). In the intestines of individuals with positive autoantibodies, the relative abundances of *Lachnospiraceae*, *Helicobacteraceae*, *Ruminococcaceae*, *Erysipelotrichaceae*, and *Bifidobacteriaceae* significantly increased (14). In our study, we also observed a significant increase in the relative abundance of *Ruminococcus*, *Lachnospirales*_unclassified, *Helicobacteraceae*, *Bifidobacterium*, *Lachnospiraceae*_NK4A136_group. *Ruminococcus* plays a significant role in digesting resistant starch, but these bacteria are also associated with intestinal diseases (IBS, IBD, Crohn’s disease, etc.), immune disorders (allergy, eczema, asthma, etc.), and neurological disorders (autism, depression, etc.) (15–19).

As the autoantibody profiles kept expanding, the disease progressed to early RA. In patients with early RA, there is a dominant gut microbiota composed mainly of *Prevotella copri*. When the microbiota of patients with early RA was transplanted into germ-free arthritis-prone SKG mice, the lymphocytes in the regional lymph nodes and colon of the mice showed a stronger response to ribosomal protein L23A, and developed severe arthritis (20). Similar results were also obtained in the study by Scher et al. (21). In the analysis of different species, we also detected the presence of *Prevotella*, but not in the CIA model group; potential underlying reasons are proposed as follows. First, technical false negatives may arise from multiple confounding factors. SPF laboratory rodents inherently carry extremely low baseline abundances of *Prevotella* in the gut; limited sequencing depth further leads to stochastic dropout of low-abundance taxa during random sampling. In addition, as an obligate anaerobe sensitive to oxygen, *Prevotella* undergoes substantial DNA degradation during repeated freeze-thaw cycles and DNA extraction procedures, collectively contributing to the failure of detection. Second, biological interspecies barriers exist in the core gut microbiota between humans and rodents. Pathogenic *P. copri*, which is enriched in patients with newly diagnosed RA, is not an indigenous gut bacterium of conventional DBA/1 mice. Consistent detection of this genus is only achievable in humanized mice or germ-free models with monocolonization of *Prevotella*. Previous CIA studies reporting elevated *Prevotella* levels all adopted supplementary interventions including human fecal microbiota transplantation or single-strain oral gavage. The absence of exogenous microbial colonization may therefore explain the undetectable *Prevotella* in naïve CIA mice without additional bacterial administration.

Patients with confirmed RA display disease-specific gut microbiota signatures. Huang et al. (22) constructed RA‐originated gut microbial biobank (RAGMB) and identified 20 core gut bacterial species constituting the intestinal core microbiota of RA patients, dominated by *Bacteroides*, *Faecalibacterium*, *Bifidobacterium*, *Eubacterium* and *Escherichia*. RAGMB covers 18 of these core species (90%), including four novel species identified in this study such as *Mediterraneibacter tenuis*. Pro-inflammatory core taxa including *Mediterraneibacter tenuis*, *Bacteroides vulgatus*, *Bacteroides ovatus* and *Escherichia coli* were significantly positively correlated with inflammatory markers ESR, CRP, TNF-α and IL-8. Statistically validated anti-inflammatory core species, such as *Eubacterium rectale*, showed negative correlations with anti-CCP antibodies, ESR, TNF-α and pro-inflammatory cytokines IL-1β, IL-6, alongside a marked positive correlation with the anti-inflammatory cytokine IL-10. Most other core taxa, such as *Faecalibacterium prausnitzii* and *Anaerostipes hadrus*, were negatively associated with multiple inflammatory biomarkers (23). In addition, other studies have supported the notion that patients with confirmed RA harbor microbiota signatures associated with RA (24, 25).

Prior preclinical and clinical investigations exploring the interplay between gut microbiota and RA have predominantly characterized intestinal microbial profiles only at a single terminal time point. In such work, gut microbial composition merely serves as an auxiliary readout alongside arthritis severity, autoantibody titers, and pro-inflammatory cytokine levels. To date, longitudinal research specifically dissecting the dynamic tripartite relationship among gut microbiota, probiotic intervention, and RA pathogenesis remains scarce. RA pathogenesis manifests as a progressive, temporally evolving pathological cascade, which is paralleled by continuous remodeling of the intestinal microbial ecosystem. Likewise, the modulatory effects exerted by *Bifidobacterium animalis* BD400 on gut commensal communities unfold in a time-dependent manner. Collectively, these lines of evidence underscore the rationality and necessity of longitudinal dynamic profiling that integrates RA disease progression, temporal gut microbial shifts, and sequential probiotic-mediated regulatory effects.

The present study implemented a longitudinal sampling strategy across multiple time points in CIA rats. We integrated alpha diversity, beta diversity and taxonomic composition analyses based on Jaccard and Bray-Curtis distances, combined with linear mixed-effects modeling to characterize temporal alterations of gut microbiota under BD400 or MTX intervention. Our findings delineate the time-resolved patterns of gut microbial succession under BD400 supplementation, and reveal the distinct microbiome features between BD400 and MTX intervention. The time-series microbial datasets generated in the present study address this critical knowledge gap within the field. By capturing continuous, multi-timepoint alterations in gut bacteria across the entire disease course, our longitudinal experimental design substantially enhances the robustness and translational reliability of the conclusions regarding BD400’s microbiota-targeted protective actions against arthritis.

## 5 Conclusion

As arthritis progresses, the gut microbiota of rats also undergoes dynamic changes. The taxa mainly involved include *Akkermansia*, *Romboutsia*, *Christensenellaceae*_R-7_group, *Bifidobacterium*, *Turicibacter*, *Candidatus*_*Saccharimonas*. Meanwhile, the influence of *B. animalis* BD400 on the gut microbiota of CIA model rats is also dynamic, mainly involving *Turicibacter*, *Bifidobacterium*, *Lactobacillus*, *Lacticaseibacillus*, *Ligilactobacillus*. In the future, we will further investigate the changes in gut microbiota metabolites as arthritis progresses.

## Data Availability

The original contributions presented in the study are publicly available. This data can be found here: The BioProject under ID PRJNA1520090, http://www.ncbi.nlm.nih.gov/bioproject/1520090.
