# Supplementary material for: The alterations in the gut microbiota and Bifidobacterium animalis BD400 during the development and progression of collagen-induced arthritis in rats
Source: Front Immunol. 2026 Sep 18;17:1821878. doi: 10.3389/fimmu.2026.1821878 (PMC13631098; doi:10.3389/fimmu.2026.1821878)
Supplement: Supplementary file 1 [file Table2.docx]

Figure S1. Evaluation of sequencing quality and sample sufficiency for gut microbiota datasets. (A) Rank‑abundance curve. (B) Rarefaction curves of observed‑species assessing sequencing depth. (C) ASV accumulation curve.

Figure S2. Temporal changes in arthritis clinical score and paw thickness in CIA rat under different treatments. (A) Arthritis clinical score; (B) Thickness of paw. Data are presented as mean ± standard deviation. **P* < 0.05, ***P* < 0.01 vs CIA model group at the same time point.

Figure.S3 Time-series PCoA plots of gut microbiota based on Jaccard and Bray-Curtis dissimilarity. PCoA plots calculated using Jaccard distance (A–I) and Bray-Curtis distance (J–R). Each dot represents an individual fecal sample, and colored ellipses represents the BD400 group (pink), the MTX group (blue), the CIA model group (purple) and the control group (yellow).
